# Behavior of Crystalline Water During Cation Adsorption and Desorption in Layered Zirconium Phosphate

**DOI:** 10.1002/cphc.70565

**Published:** 2026-10-15

**Authors:** Masakuni Yamaguchi, Takahiro Toyoshima, Akira Takahashi, Tohru Kawamoto, Hisashi Tanaka

**Affiliations:** ^1^ Resource Circulation Adsorption Research Group Research Institute of Core Technology for Materials Innovation Department of Materials and Chemistry National Institute of Advanced Industrial Science and Technology Tsukuba Ibaraki Japan

**Keywords:** alkali ion removal, alkaline wastewater, ammonium adsorption, ion exchange, layered phosphate

## Abstract

This study explores the behavior of crystalline water during the adsorption of Na^+^, K^+^, and NH_4_^+^ ions onto layered zirconium phosphate (ZrP) using powder X‐ray diffraction (XRD) and thermogravimetric analysis (TG). Cation uptake proceeded via two discrete adsorption steps, each accompanied by distinct structural transformations and water‐related phenomena. For Na^+^, water molecules were incorporated into the interlayer region during the second adsorption stage. In contrast, adsorption of K^+^ and NH_4_^+^ involved the release of crystalline water during the first stage, followed by water incorporation in the second stage. Notably, ZrP exhibited no measurable dissolution under the analytical conditions employed even under strongly alkaline conditions (pH ≈ 12). Furthermore, acid‐induced desorption followed by readsorption confirmed that the layered structure of ZrP remained intact and fully regenerable. These findings demonstrate that ion‐dependent migration of crystalline water plays a critical role in the adsorption mechanism and indicate that the dynamic behavior of interlayer water must be considered in the rational design of layered inorganic materials.

## Graphical abstract

Crystalline water behavior during Na^+^, K^+^, and NH_4_^+^ adsorption onto layered zirconium phosphate (ZrP) was investigated. Cation uptake occurred through a two‐step mechanism involving ion‐dependent release and incorporation of interlayer water. These findings demonstrate that crystalline water migration and redistribution critically govern cation adsorption and structural transformations within the layered material system.

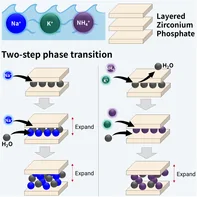

## 1 Introduction

Layered zirconium phosphate (Zr(HPO_4_)_2_·H_2_O; ZrP) is an inorganic layered compound characterized by a stable and well‐defined interlayer structure and high chemical stability [1, 2, 3]. Because ZrP contains exchangeable protons (H^+^) within its interlayers, it has been extensively investigated as a solid acid and as a versatile platform for interlayer reactions [4, 5, 6]. In catalysis, its strong Brønsted acidity has been exploited for esterification and hydrolysis, and it has also served as a heterogeneous catalyst support via intercalation of metal complexes or nanoparticles [7, 8, 9]. Moreover, owing to its relatively high in‐plane cation conductivity, layered ZrP has attracted considerable attention for applications in high‐sensitivity impedance‐type sensors and as a solid electrolyte for intermediate‐temperature fuel cells [10, 11, 12]. These functionalities are closely associated with the nature of interlayer cation species and the presence and state of crystalline water [13, 14]. Layered inorganic ion exchangers have attracted increasing attention for the removal and recovery of ionic species from aqueous streams. In particular, the selective recovery of ammonium and alkali metal ions is relevant to wastewater treatment, nutrient recycling, and sustainable resource management [1, 2, 3, 4, 5, 6]. Ammonium is not only a major contaminant in agricultural and municipal wastewaters but also a potentially valuable nitrogen resource. Consequently, understanding the mechanisms governing ion uptake in inorganic ion exchangers is essential for improving separation performance and enabling efficient resource recovery.

Layered ZrP is also widely recognized as an inorganic ion exchanger capable of capturing a variety of cations [15, 16, 17]. It is generally accepted that exchanging cations with high hydration energy, such as sodium ions (Na^+^), induces significant interlayer expansion, commonly referred to as swelling [10]. This expansion has been attributed to the incorporation of substantial amounts of hydration water into the interlayer region during high‐density cation exchange. Accordingly, the prevailing view holds that cation introduction is accompanied by an increase or retention of interlayer water molecules and an expansion of the interlayer spacing. However, the detailed reaction pathways, intermediate states, and the precise role of crystalline water during multistep adsorption processes remain poorly understood. Crystalline water plays an important role in layered inorganic materials, as it strongly influences ion transport, structural stability, swelling behavior, and ion‐exchange properties [10, 11, 12, 13]. In ZrP and related layered compounds, cation exchange is generally accompanied by changes in interlayer spacing and hydration state. Previous structural studies have reported hydrated ion‐exchanged phases such as sodium‐ and potassium‐exchanged ZrPs, suggesting that water molecules help stabilize the exchanged structures [18, 22]. However, most previous investigations have focused on the structures before and after ion exchange, whereas the evolution of crystalline water during the ion‐exchange process itself has rarely been examined in detail. Despite the existence of several hydrated ion‐exchanged ZrP phases, the behavior of crystalline water during stepwise ion exchange remains poorly understood. In particular, it is unclear whether crystalline water is retained, released, or incorporated during each stage of ion exchange and how this behavior depends on the exchanged cation species. This unresolved issue limits the mechanistic understanding of ion selectivity in layered ion exchangers.

In this study, changes in the amount of crystalline water during the uptake of Na^+^, K^+^, and NH_4_^+^ ions by layered ZrP were investigated using powder X‐ray diffraction (XRD) and thermogravimetric analysis (TG). By comparing structural evolution and volatile‐species behavior at different stages of ion exchange, the role of crystalline water during stepwise cation exchange was clarified. These findings provide new insight into ion‐dependent exchange mechanisms in layered inorganic materials and emphasize the importance of considering crystalline water dynamics in the rational design of separation and resource‐recovery materials.

## 2 Experimental Methods

### 2.1 Material Preparation

ZrP was synthesized by refluxing zirconium oxide with phosphoric acid at 473 K for 24 h. The resulting solid was collected by filtration, repeatedly washed with Milli‐Q water, and vacuum‐dried at room temperature. The synthesized material was characterized by powder XRD and TG. Detailed synthesis conditions are provided in Supporting Information: Section 1.1.

### 2.2 Adsorption Experiments

The adsorption behaviors of Na^+^, K^+^, and NH_4_^+^ ions on ZrP were investigated using batch adsorption experiments. Approximately 600 mg of ZrP was suspended in 30 mL of cation solution (solid‐to‐liquid ratio: 50 mL g^−1^) and stirred for 6 h at temperatures between 283 and 333 K. After adsorption, the suspension was filtered through a 0.45 μm membrane filter, and the recovered solids were washed with Milli‐Q water and vacuum‐dried before characterization. Adsorption capacities were calculated from the concentration differences before and after adsorption. Detailed experimental procedures are provided in Supporting Information: Section 1.2.

### 2.3 Solution Analysis

The concentrations of Na^+^, K^+^, and NH_4_^+^ ions were determined by ion chromatography (IC, Eco IC, Metrohm). Dissolved zirconium and phosphorus concentrations were measured by inductively coupled plasma mass spectrometry (ICP–MS, NexION 2000, PerkinElmer). The limits of quantification were 4 ppb for ^90^Zr and 40 ppb for ^31^P. Detailed analytical conditions, calibration methods, and instrumental parameters are provided in Supporting Information: Section 1.3.

### 2.4 Solid‐State Characterization

Powder XRD (D8 Advance, Bruker) analysis using Cu Kα radiation at 40 kV and 40 mA was used to evaluate structural changes in the layered materials. Thermogravimetric and differential thermal analyses (TG–DTA, Thermo Plus EVO2 TG‐DTA8122, Rigaku) were conducted under a nitrogen atmosphere at a heating rate of 5 K min^−1^. Elemental compositions were analyzed using scanning electron microscopy coupled with energy‐dispersive X‐ray spectroscopy (SEM–EDX, TM4000Pro, Hitachi High‐Tech). Additional measurement conditions are described in Supporting Information: Section 1.4.

### 2.5 Regeneration Experiments

The reversibility of cation adsorption was evaluated through acid desorption and subsequent readsorption experiments. Cation‐loaded ZrP samples were treated with hydrochloric acid solution, then washed, dried, and readsorbed using the corresponding cation solutions. The regenerated materials were characterized by XRD, TG–DTA, SEM–EDX, and ICP–MS analyses. Detailed regeneration procedures are provided in Supporting Information: Section 1.5.

## 3 Results and Discussion

### 3.1 Cation Adsorption Performance on Layered ZrP

Figure 1 presents the Na^+^, K^+^ and NH_4_^+^ adsorption isotherms of ZrP measured at 283, 298, 313, and 333 K. For each cation species, two distinct regions are observed at all temperatures, in which the equilibrium concentration remains nearly constant while the amount of adsorbed cation varies. These constant‐concentration domains, hereafter referred to as plateau regions, indicate a stepwise adsorption process. In this study, the lower‐loading plateau is defined as Region A (0–3.4 mmol_cation_ g_ZrP_^−1^, corresponding to 0–1 mol_cation_ mol_ZrP_^−1^), whereas the higher‐loading plateau is defined as Region B (3.4–6.7 mmol_cation_ g_ZrP_^−1^, corresponding to 1–2 mol_cation_ mol_ZrP_^−1^). A comparable two‐step adsorption profile has previously been reported for ammonia uptake in layered phosphate systems [18], suggesting that such materials exhibit adsorption mechanisms characterized by discrete equilibrium states. Based on the reaction scheme proposed for ammonia adsorption, the cation‐exchange process under acidic or neutral pH conditions may be represented by Equations (I) and (II):

**FIGURE 1:**
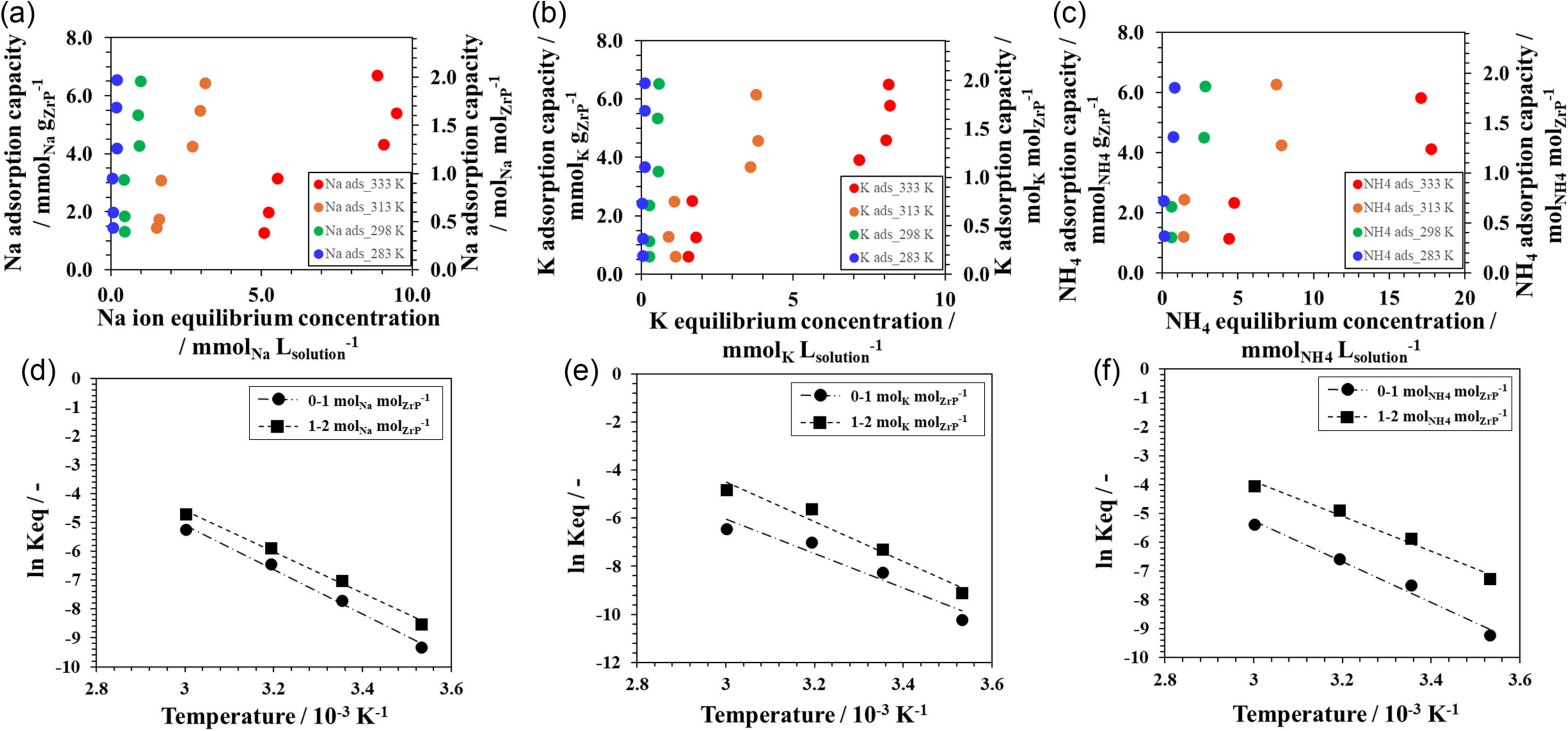
Cation adsorption performance of ZrP for Na^+^, K^+^, and NH_4_^+^ at 283–333 K, and the corresponding van’t Hoff plot. (a–c) adsorption results and (d–f) van’t Hoff plot.

(I) $$\mathrm{ZrP}-2H^{+}+A^{+}⇌\mathrm{ZrP}-H^{+}A^{+}+H^{+}$$

(II) $$\mathrm{ZrP}-H^{+}A^{+}+A^{+}⇌\mathrm{ZrP}-2A^{+}+H^{+}$$

where A^+^ represents one of the monovalent cations Na^+^, K^+^, or NH_4_^+^. These equations are intended only to illustrate the overall proton–cation exchange stoichiometry. Cation‐dependent structural changes, including the incorporation or release of crystalline water, are omitted for simplicity.

Assuming that the van’t Hoff equation appropriately describes the temperature dependance of the equilibrium constant *K*_eq_ for reactions (I) and (II),

(1) $$\ln K_{\mathrm{eq}}=-\frac{\text{Δ}H^{\circ }}{RT}+\frac{\text{Δ}S^{\circ }}{R}$$

the standard enthalpy change (Δ*H*°) and standard entropy change (Δ*S*°) can be determined. *R* is the universal gas constant and *T* is the absolute temperature. In this study, the equilibrium constant at each temperature was approximated as the ratio of equilibrium cation concentration *C*_eq_ to the standard reference concentration (*C*_0_ = 1M), yielding the following expression [19]:

(2) $$\ln\frac{C_{\mathrm{eq}}}{C_{0}}=-\frac{\text{Δ}H^{\circ }}{RT}+\frac{\text{Δ}S^{\circ }}{R}$$

Figure 1d shows the van’t Hoff plots for the first‐stage reaction (Region A) and the second‐stage reaction (Region B) for each cation‐exchange system. In all cases, the plots of ln *C*_eq_ versus 1/*T* yielded linear relationships, confirming that the adsorption process at each stage conformed well to the van’t Hoff behavior.

The values of Δ*H*° and Δ*S*° derived from Equation (2) for ZrP and each cation species (K^+^, Na^+^, and NH_3_) in the respective adsorption regions are summarized in Table 1. Examination of the two‐step adsorption process reveals that Na^+^ and K^+^ exhibit highly similar Δ*H*° values in both stages. For Na^+^ adsorption, the enthalpy change (Δ*H*° = 60–68 kJ mol^−1^) is close to the reported standard enthalpy difference between NaHSO_4_ and Na_2_SO_4_ (approximately 55 kJ mol^−1^).

**TABLE 1:** Standard thermodynamic parameters for adsorption on ZrP.

	Na^+^		K^+^		NH_4_^+^	
	Δ*H*º, kJmol _Na_^−1^	Δ*S*º, JK^−1^mol_Na_^−1^	Δ*H*º, kJmol_K_^−1^	Δ*S*º, JK^−1^mol_K_^−1^	Δ*H*º, $\mathrm{kJ}{{\mathrm{mol}}_{{\mathrm{NH}}_{4}}}^{‐1}$	Δ*S*º, ${\mathrm{JK}}^{‐1}{{\mathrm{mol}}_{{\mathrm{NH}}_{4}}}^{‐1}$
First‐stage adsorption	64.0	−150	59.6	−129	58.9	−133
Second‐stage adsorption	59.5	−140	68.7	−169	50.3	−119

This similarity suggests that reactions of comparable energetic magnitude occur within the layered phosphate framework. These results further indicate that moderately strong and consistent interactions between the exchanged cations and the host lattice are maintained throughout both adsorption stages.

These thermodynamic characteristics indicate that immersion in a strongly acidic solution can effectively reverse adsorption in both regions, thereby enabling efficient desorption and recovery of cations from ZrP.

The solution pH before and after cation adsorption on ZrP was examined to elucidate the proton–cation exchange behavior. At a solid–liquid ratio of 50, pronounced pH shifts were observed in both adsorption regions. In the NaOH aqueous system, the pH in Region A decreased from an initial value of 11.6–11.9 to 5.3–5.8 after adsorption. In Region B, the pH decreased from 12.0–12.1 to approximately 7.2–8.2. When the alkalinity of the starting solution exceeded these values, the pH remained largely unchanged during adsorption. In the KOH system, Region A exhibited a pH decrease from 11.4–11.9 to 4.4–4.6 after adsorption, while Region B showed a decrease from 12.0–12.2 to approximately 7.7–8.6. Similarly, highly concentrated alkaline solutions resulted in minimal pH variation before and after adsorption. For the NH_4_OH system, the pH in Region A decreased from 10.8–11.2 to 5.6–7.4 following adsorption, and Region B exhibited a decrease from 11.1–11.3 to 7.2–7.5. These significant pH decreases indicate that protons are released upon cation uptake, consistent with the ion‐exchange reactions proposed in Equations (I) and (II). Therefore, the uptake process observed in the present study should be regarded as proton–cation ion exchange rather than simple surface adsorption. In this manuscript, the term “adsorption” is retained only to describe the overall uptake behavior, adsorption isotherms, and adsorption capacities.

To assess whether ZrP dissolved during adsorption, the concentrations of dissolved ^90^Zr and ^31^P in the supernatant were quantified using ICP–MS. Because high concentrations of Na^+^ or K^+^ can destabilize the plasma, each supernatant was diluted prior to analysis, up to a maximum dilution factor of 100. As shown in Figure S1, the instrumental detection limits for the diluted samples were 5 ppb for ^90^Zr and 50 ppb for ^31^P. In all measurements, the concentrations of ^90^Zr and ^31^P in the diluted samples were below these detection limits. When converted to concentrations in the original undiluted solutions, these values correspond to less than 500 ppb for ^90^Zr and less than 5000 ppb for ^31^P. The true concentrations are likely lower depending on the dilution factor applied to each sample. The detection limits correspond to less than 0.008% of the total Zr and less than 0.12% of the total P initially present in the solid phase, even assuming the maximum dilution factor. Therefore, any dissolution of ZrP, if present, was negligible under the experimental conditions employed. These results demonstrate that no measurable dissolution of ZrP occurred under any of the adsorption conditions examined.

### 3.2 Physicochemical Changes in ZrP Before and After Cation Adsorption

To verify structural changes in layered ZrP during cation adsorption, powder XRD measurements were performed on samples before and after adsorption. The objective was to determine whether distinct crystallographic transformations accompanied the expected two‐step adsorption process. Previous studies have reported that both pristine Zr(HPO_4_)_2_·H_2_O and K^+^‐containing Zr(K_2_PO_4_)_2_·H_2_O exhibit well‐defined layered structures [20, 21]. In addition, for Na^+^, the existence of a two‐dimensional hydrated phase, Zr_2_(NaPO_4_)_4_·3H_2_O, partially coordinated by water molecules, has also been documented [22].

Figure 2 presents the XRD patterns of ZrP before and after cation adsorption at different loading levels. The three patterns in the upper row correspond to samples in which the second‐stage reaction is presumed to have occurred, while the three patterns in the middle row correspond to samples attributed to the first‐stage reaction. The bottom pattern represents pristine ZrP before cation adsorption.

**FIGURE 2:**
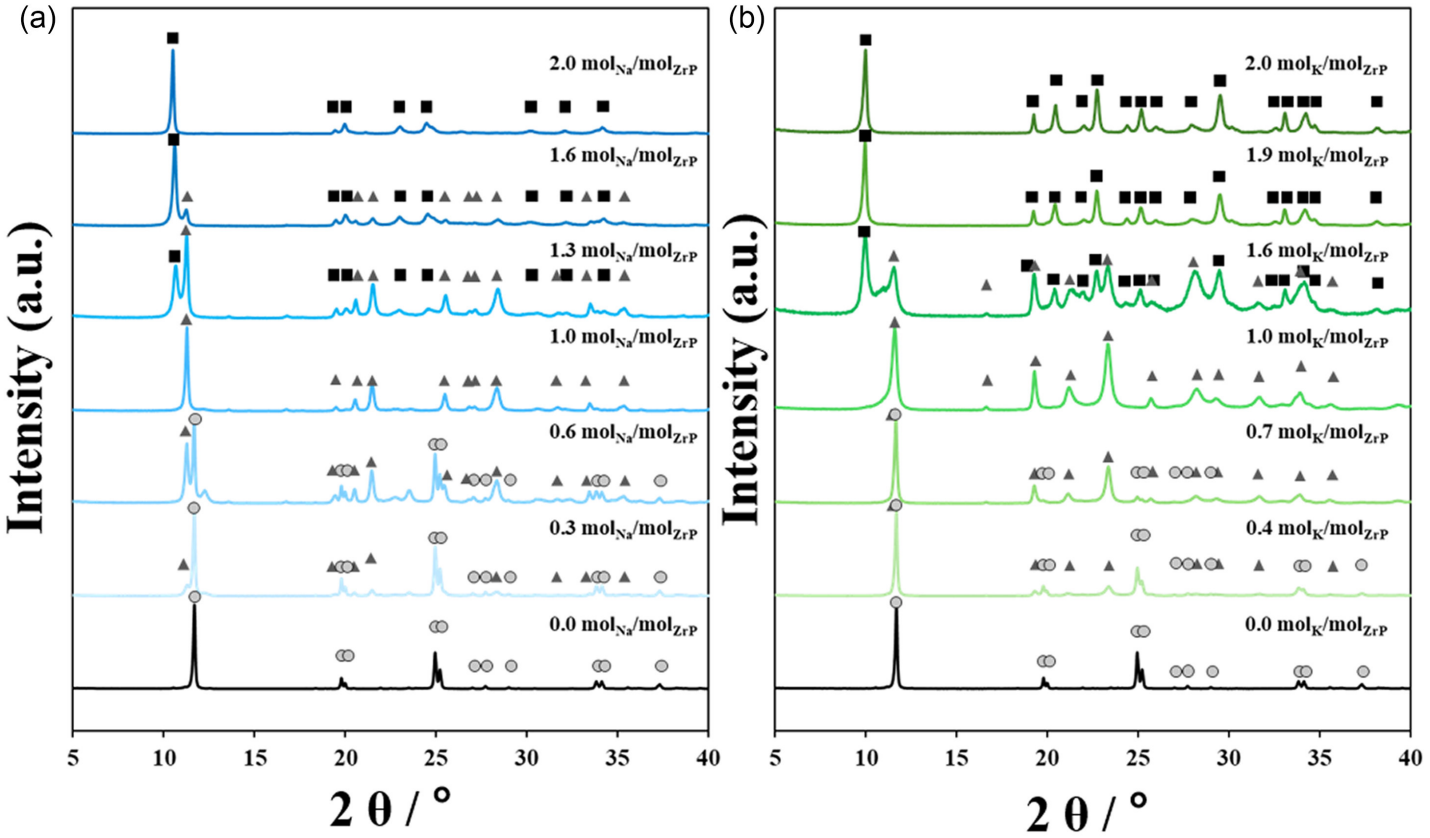
XRD patterns of ZrP after cation adsorption: (a) Na^+^‐adsorbed ZrP at various adsorption amounts and (b) K^+^‐adsorbed ZrP at various adsorption amounts. The diffraction patterns are vertically offset for clarity. Symbols indicate different structural states: ●, pristine ZrP; ▲, presumed to represent ZrP with 1 mol_cation_/mol_ZrP_; and ◼, presumed to represent ZrP with 2 mol_cation_/mol_ZrP_.

For the samples assigned to the second‐stage reaction, noticeable changes in peak intensities and positions were observed. These diffraction features are consistent with the reported crystalline phases Zr_2_(NaPO_4_)_4_·3H_2_O and Zr(K_2_PO_4_)_2_·H_2_O, suggesting that these phases were newly formed or partially manifested during the adsorption process. In contrast, a distinct, previously unreported diffraction pattern was observed for the sample associated with the first‐stage reaction (fourth pattern from the top). The sharpness and resolution of the peaks indicate the formation of a new, well‐defined crystalline phase rather than a poorly ordered intermediate.

Notably, for K^+^‐adsorbed ZrP, the diffraction peak near 2*θ* ≈ 12°, which reflects the interlayer spacing, exhibited no significant shift. This suggests that structural modification during the first‐stage K^+^ adsorption occurs predominantly within the intralayer (in‐plane) framework rather than along the stacking direction. In contrast, the Na^+^‐adsorbed sample showed a clear shift of the same diffraction peak, indicating that Na^+^ incorporation perturbs not only the in‐plane framework but also the stacking direction, i.e., the interlayer arrangement. The occurrence of distinct adsorption plateaus together with the appearance of characteristic XRD patterns suggests that the cation uptake process does not proceed through a continuous structural evolution. Instead, specific structural states appear at approximately 1 and 2 mol_cation_ mol_ZrP_^−1^, corresponding to the two plateau regions observed in Figure 1. Although direct in situ structural characterization was not performed, the adsorption equilibria and XRD phase evolution are consistent with a stepwise structural transformation during cation uptake.

To investigate the behavior of crystalline water and other volatile components in ZrP during cation adsorption, TG–DTA was performed. In the temperature range examined, the observed mass loss primarily arises from the evolution of crystalline water (H_2_O). The fractional mass loss *W*_loss_ was defined as:

(3) $$W_{\text{loss}}=\frac{W_{H_{2}O}}{W_{\text{sample},\text{before}}}$$

where $W_{H_{2}O}$ is the mass of released water and *W*_sample, before_ is the initial sample mass prior to dehydration. The molar release of water per formula unit (*M*_loss_) was calculated as:

(4) $$M_{\text{loss}}=W_{\text{loss}}\frac{M_{\mathrm{ZrP}}}{M_{H_{2}O}}$$

where $M_{H_{2}O}$ and *M*_ZrP_ are the molar masses of water and ZrP, respectively. Although DTA signals were simultaneously recorded, the present discussion focuses primarily on TG mass‐loss behavior because the thermal effects were weak and difficult to interpret reliably.

No distinct low‐temperature mass‐loss step attributable to physically adsorbed water was observed [18]. The cation exchange ratio of each sample was determined from the experimentally measured cation uptake. Based on this ratio, the average composition of the partially exchanged phase was estimated and used to convert the TG mass loss into volatile species per mole of ZrP. Figure 3 shows the TG curves for pristine ZrP and the samples after Na^+^, K^+^, and NH_4_^+^ adsorption. The mass loss is expressed in units of ${\mathrm{mol}}_{H_{2}O}$ mol _ZrP_^−1^.

For the first‐stage products (Figure 3a), the net mass loss remains essentially unchanged compared with pristine ZrP, although the dehydration temperature shifts to higher values. This behavior indicates strengthened interactions between framework water and the Na^+^ introduced into the structure. In contrast, the second‐stage products (Figure 3dc) show a gradual increase in mass loss. At a loading of 2 mol_Na_ mol_ZrP_^−1^, the total mass loss corresponds to $\approx 1.5\;{\mathrm{mol}}_{H_{2}O}$ mol _ZrP_^−1^. These results are consistent with stepwise transformation toward the hydrated sodium phase Zr_2_(NaPO_4_)_4_·3H_2_O.

**FIGURE 3:**
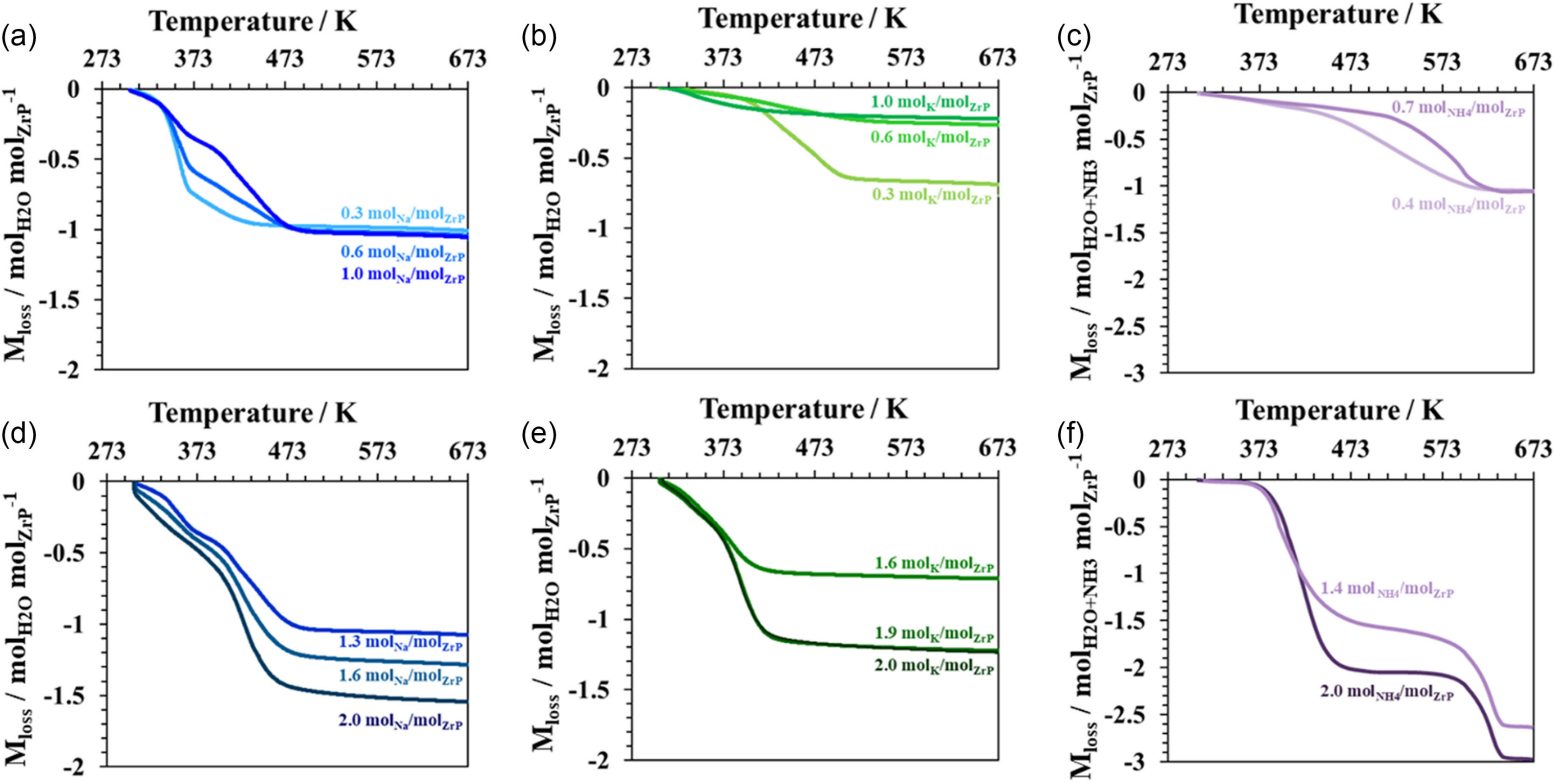
TG profiles of ZrP with different Na^+^ and K^+^ adsorption amounts: first‐stage adsorption area of (a) Na^+^, (b) K^+^, and (c) NH_4_^+^, and second‐stage adsorption area of (d) Na^+^, (e) K^+^, and (f) NH_4_^+^.

Accordingly, the dehydration behavior of the Na^+^ system under TG–DTA can be represented by the following scheme (0 ≤ *x* ≤ 1 and 0 ≤ *y* ≤ 1):

(III) $$\begin{array}{l} \left(1-x\right)\mathrm{Zr}{\left({\mathrm{HPO}}_{4}\right)}_{2}\cdot H_{2}O+x\mathrm{Zr}\left({\mathrm{NaPO}}_{4}\right)\left({\mathrm{HPO}}_{4}\right)\cdot H_{2}O\\ \;\to (1-x)\mathrm{Zr}{\left({\mathrm{HPO}}_{4}\right)}_{2}+\mathrm{xZr}({\mathrm{NaPO}}_{4})({\mathrm{HPO}}_{4})+H_{2}O \end{array}$$

(IV) $$\begin{array}{l} \begin{array}{l} (1-y)\mathrm{Zr}({\mathrm{NaPO}}_{4})({\mathrm{HPO}}_{4})\cdot H_{2}O+y\mathrm{Zr}{\left({\mathrm{NaPO}}_{4}\right)}_{2}\cdot 3/2H_{2}O\\ \;\to (1-y)\mathrm{Zr}({\mathrm{NaPO}}_{4})({\mathrm{HPO}}_{4})+\mathrm{yZr}{\left({\mathrm{NaPO}}_{4}\right)}_{2}\\ \;+(1+1/2y)H_{2}O \end{array} \end{array}$$

For the first‐stage products (Figure 3b), the total mass loss decreases with increasing K^+^ uptake, indicating that K^+^ exchange is accompanied by the release of crystalline water from ZrP. In contrast, the second‐stage products (Figure 3e) exhibit an increase in mass loss, with a final value corresponding to $\sim 1.0\;{\mathrm{mol}}_{H_{2}O}$ mol _ZrP_^−1^. These features suggest that the second stage involves the coincorporation of water with K^+^, consistent with the formation of the hydrated phase Zr(KPO_4_)_2_·H_2_O and a progressive hydration mechanism.

The corresponding TG–DTA dehydration scheme for the K^+^ system is thus (0 ≤ *x *≤ 1 and 0 ≤ *y *≤ 1):

(V) $$\begin{array}{l} \left(1-x)\mathrm{Zr}{\left({\mathrm{HPO}}_{4}\right)}_{2}\cdot H_{2}O+x\mathrm{Zr}({\mathrm{KPO}}_{4})({\mathrm{HPO}}_{4}\right)\\ \;\to \;(1-x)\mathrm{Zr}{\left({\mathrm{HPO}}_{4}\right)}_{2}+x\mathrm{Zr}({\mathrm{KPO}}_{4})({\mathrm{HPO}}_{4})+(1-x)H_{2}O \end{array}$$

(VI) $$\begin{array}{l} (1-y)\mathrm{Zr}({\mathrm{KPO}}_{4})({\mathrm{HPO}}_{4})+y\mathrm{Zr}{\left({\mathrm{KPO}}_{4}\right)}_{2}\cdot H_{2}O\\ \;\to \;(1-y)\mathrm{Zr}({\mathrm{KPO}}_{4})({\mathrm{HPO}}_{4})+\mathrm{yZr}{\left({\mathrm{KPO}}_{4}\right)}_{2}+{\mathrm{yH}}_{2}O\; \end{array}$$

For the first‐stage products (Figure 3c), the overall mass change is small, but the temperature window for volatilization shifts. Because crystalline water (MW = 18) and ammonia (MW = 17) evolve over overlapping temperature ranges, their combined mass loss is detected by TG. The data suggests that, during the first stage, the amount of crystalline water expelled is approximately equimolar to the amount of NH_4_^+^ introduced. In the second‐stage products (Figure 3f), a larger mass loss is observed, corresponding to $\sim 3\;{\mathrm{mol}}_{H_{2}O}+{\mathrm{NH}}_{3}$ mol _ZrP_^−1^. These results indicate coincorporation of NH_4_^+^ and water in the second stage, consistent with the formation of Zr(NH_4_PO_4_)_2_·H_2_O, and a progressive hydration mechanism. The observed mass loss is consistent with the release of volatile species such as crystalline water and/or ammonia during NH_4_^+^ uptake. Although TG–DTA alone does not allow direct discrimination between H_2_O and NH_3_, a previous TG–MS study of the fully exchanged phase Zr(NH_4_PO_4_)_2_·H_2_O reported the release of approximately one mole of H_2_O and one mole of NH_3_ below 473 K, followed by the release of an additional mole of NH_3_ at higher temperatures [18]. The present TG–DTA behavior is consistent with this reported decomposition pathway.

The corresponding TG–DTA dehydration scheme for the NH_4_^+^ system is thus (0 ≤ *x* ≤ 1 and 0 ≤ *y* ≤ 1):

(VII) $$\begin{array}{l} \left(1-x)\mathrm{Zr}{\left({\mathrm{HPO}}_{4}\right)}_{2}\cdot H_{2}O+x\mathrm{Zr}({\mathrm{NH}}_{4}{\mathrm{PO}}_{4})({\mathrm{HPO}}_{4}\right)\\ \;\to \;\mathrm{Zr}{\left({\mathrm{HPO}}_{4}\right)}_{2}+(1-x)H_{2}O+x{\mathrm{NH}}_{3} \end{array}$$

(VIII) $$\begin{array}{l} \left(1-y\right)\mathrm{Zr}\left({\mathrm{NH}}_{4}{\mathrm{PO}}_{4}\right)\left({\mathrm{HPO}}_{4}\right)+y\mathrm{Zr}{\left({\mathrm{NH}}_{4}{\mathrm{PO}}_{4}\right)}_{2}\cdot H_{2}O\\ \;\to \;\mathrm{Zr}{\left({\mathrm{HPO}}_{4}\right)}_{2}+{\mathrm{yH}}_{2}O+\left(1+y\right){\mathrm{NH}}_{3} \end{array}$$

Notably, the assignment of H_2_O and NH_3_ emission in the NH_4_^+^ system is based on a combined interpretation of adsorption stoichiometry, XRD phase evolution, TG–DTA behavior, and previously reported TG–MS data [18]. Based on the above results, the cation adsorption processes for layered ZrP can be summarized as:

(IX) $$\begin{array}{l} \mathrm{Zr}{\left({\mathrm{HPO}}_{4}\right)}_{2}\cdot H_{2}O+{\mathrm{Na}}^{+}{\mathrm{OH}}^{-}⇌\mathrm{Zr}({\mathrm{NaPO}}_{4})({\mathrm{HPO}}_{4})\cdot H_{2}O+H_{2}O \end{array}$$

(X) $$\begin{array}{l} \mathrm{Zr}({\mathrm{NaPO}}_{4})({\mathrm{HPO}}_{4})\cdot H_{2}O+{\mathrm{Na}}^{+}{\mathrm{OH}}^{-}\\ \;⇌\mathrm{Zr}{\left({\mathrm{NaPO}}_{4}\right)}_{2}\cdot 3/2H_{2}O+1/2H_{2}O \end{array}$$

(XI) $$\begin{array}{l} \mathrm{Zr}{\left({\mathrm{HPO}}_{4}\right)}_{2}\cdot H_{2}O+K^{+}{\mathrm{OH}}^{-}\\ \;⇌\mathrm{Zr}({\mathrm{KPO}}_{4})({\mathrm{HPO}}_{4})+2H_{2}O \end{array}$$

(XII) $$\mathrm{Zr}({\mathrm{KPO}}_{4})({\mathrm{HPO}}_{4})+K^{+}{\mathrm{OH}}^{-}⇌\mathrm{Zr}{\left({\mathrm{KPO}}_{4}\right)}_{2}\cdot H_{2}O$$

(XIII) $$\mathrm{Zr}{\left({\mathrm{HPO}}_{4}\right)}_{2}\cdot H_{2}O+{{\mathrm{NH}}_{4}}^{+}{\mathrm{OH}}^{-}⇌\mathrm{Zr}({\mathrm{NH}}_{4}{\mathrm{PO}}_{4})({\mathrm{HPO}}_{4})+2H_{2}O$$

(XIV) $$\mathrm{Zr}({\mathrm{NH}}_{4}{\mathrm{PO}}_{4})({\mathrm{HPO}}_{4})+{{\mathrm{NH}}_{4}}^{+}{\mathrm{OH}}^{-}⇌\mathrm{Zr}{\left({\mathrm{NH}}_{4}{\mathrm{PO}}_{4}\right)}_{2}\cdot H_{2}O$$

### 3.3 Regeneration of ZrP via Acid Desorption and Subsequent Readsorption

To evaluate the renderability of layered ZrP, each cation‐adsorbed ZrP sample underwent acid desorption with hydrochloric acid, followed by readsorption of the same cation. As shown in Figure 4, the XRD pattern of the acid‐treated material was identical to that of pristine ZrP, demonstrating that the original layered structure was fully restored and that the acid treatment effectively removed the previously adsorbed cations.

**FIGURE 4:**
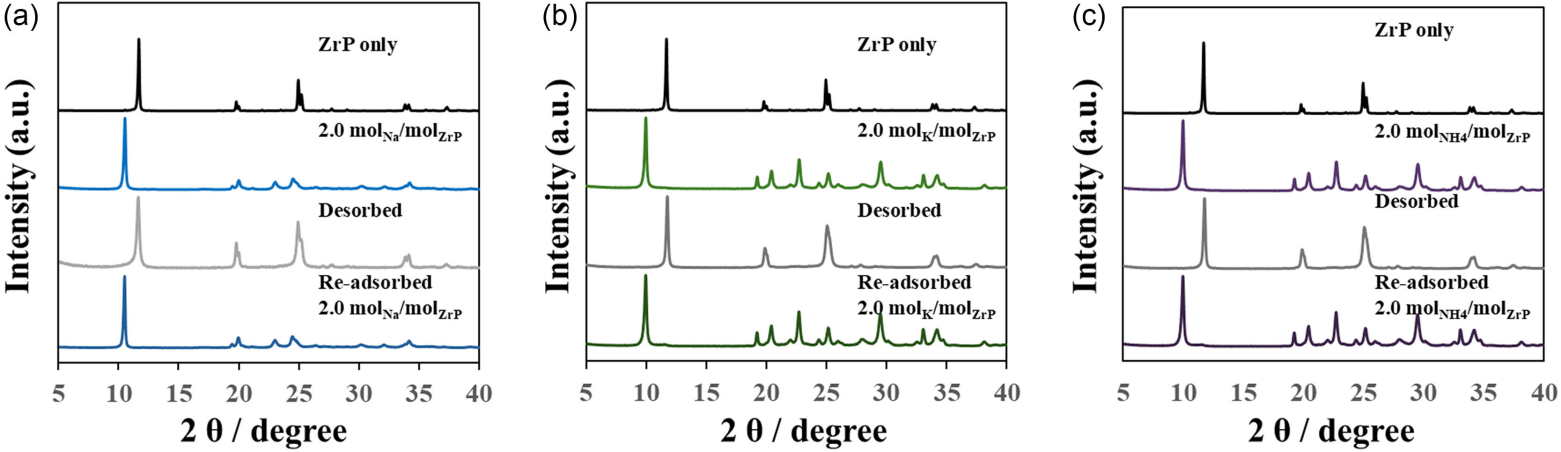
XRD patterns of cation‐adsorbed ZrP after acid desorption and subsequent readsorption of (a) Na^+^, (b) K^+^, and (c) NH_4_^+^.

Moreover, the XRD pattern obtained after the subsequent readsorption step reproduced the diffraction pattern features observed during the initial adsorption process. This result indicates that repeated adsorption–desorption cycles did not induce structural degradation of the ZrP framework and that the ion‐exchange process is reversible.

Additional SEM–EDX analysis showed that the Na/P and K/P molar ratios decreased to nearly zero after acid desorption and returned to values close to those of the original exchanged samples after readsorption (Table S2), further supporting the reversible uptake and release of exchangeable cations within the ZrP structure.

The pH of the filtrate after readsorption was approximately 12 across all systems, showing minimal deviation from the pH of the initial adsorption solutions. Additionally, ICP–MS analysis of the filtrate confirmed that the concentrations of dissolved Zr and P remained below their respective detection limits. These findings demonstrate that the regenerated ZrP did not undergo measurable dissolution during either the acid treatment or the subsequent readsorption process. These adsorption–desorption–readsorption experiments provide direct evidence that the ion‐exchange process is reversible and that the layered framework remains stable during regeneration.

## 4 Conclusion

This study systematically investigated the behavior of crystalline water during the adsorption of Na^+^, K^+^, and NH_4_^+^ ions onto layered ZrP using powder XRD and TG–DTA. The results demonstrate that cation uptake proceeds via a distinct two‐step mechanism, with each stage accompanied by characteristic structural transformations and water‐related phenomena.

For Na^+^ adsorption, the first stage involved negligible loss of crystalline water, whereas the second stage indicated the co‐incorporation of water molecules together with the cation. In contrast, K^+^ adsorption exhibited substantial release of crystalline water during the first stage, followed by rehydration in the second stage as water molecules were introduced into the interlayer structure along with K^+^. For NH_4_^+^ adsorption, the first stage involved desorption of crystalline water, approximately equal to the amount of introduced cations. In contrast, the second stage showed simultaneous uptake of water and ammonia in comparable molar quantities. These findings reveal that the dynamic behavior of crystalline water differs markedly among cation species and adsorption stage, underscoring the critical role of water in stabilizing the interlayer structure and governing the functional properties of layered ZrP.

Additionally, desorption and subsequent readsorption experiments using acid treatment confirmed that ZrP can be regenerated without structural degradation. The complete recovery of the original XRD pattern and the absence of detectable Zr or P dissolution demonstrate the material’s structural robustness and reusability under both acidic and alkaline conditions. Overall, this study provides mechanistic insights into the ion‐dependent dynamics of crystalline water in layered ZrP. These results contribute to a deeper understanding of ion selectivity in layered inorganic materials and offer guidance for the rational design of high‐performance separation and functional materials by controlling interlayer crystalline water.

## Funding

This research received no external funding.

## Conflicts of Interest

The authors declare that they have no conflicts of interest.

## Supporting information

Supplementary Material

## Data Availability

The data that support the findings of this study are available from the corresponding author upon reasonable request.
