# Supplementary material for: Behavior of Crystalline Water During Cation Adsorption and Desorption in Layered Zirconium Phosphate
Source: Chemphyschem. 2026 Oct 15;27(20):e70565. doi: 10.1002/cphc.70565 (PMC13671877; doi:10.1002/cphc.70565)
Supplement: Supplementary file 1 [file CPHC-27-e70565-s001.pdf]

Supporting Information

BEHAVIOR OF CRYSTALLINE WATER DURING CATION ADSORPTION AND DESORPTION IN LAYERED ZIRCONIUM PHOSPHATE

Masakuni Yamaguchi[a], Takahiro Toyoshima[a], Akira Takahashi[a], Tohru Kawamoto[a], and Hisashi Tanaka[a]

Corresponding author: hisashi.tanaka@aist.go.jp

Supplementary Materials Contents

Materials and methods

1.1 Material Preparation	2
1.2 Adsorption Batch Experiments	2
1.3. Solution Property Characterization	3
1.3.1. Ion chromatography (IC)	3
1.3.2. Inductively coupled plasma mass spectrometer (ICP-MS)	3
1.4 Solid-State Characterization	3
1.4.1 Powder X-ray Diffraction (XRD)	4
1.4.2 Thermogravimetric and Differential Thermal Analysis (TG-DTA)	4
1.4.3 Scanning Electron Microscopy coupled with Energy-Dispersive X-ray Spectroscopy (SEM-EDX)	4
1.5 Acid Regeneration and Re-adsorption of ZrP	4
Figure S1 Calculation curves of ICP-MS using ^{115}In, (a)^{90}Zr and (b) ^{31}P	5
Table S1. Calibration parameters for ICP-MS analysis of ^{90}Zr and ^{31}P.	5
Table S2. Na/P and K/P molar ratios determined by SEM-EDX.	5

1. Materials and methods

1.1 Material preparation

Layered zirconium phosphate (ZrP) was synthesized via a reflux method using zirconium oxide (ZrO_2, FUJIFILM Wako Pure Chemical Corporation, Osaka, Japan, ≥99%) and phosphoric acid (H_3PO_4, Nacalai Tesque, Inc., Kyoto, Japan, 85%). Zirconium oxide (5.00 g) was added to 50 mL of H_3PO_4, and the mixture was heated to 473 K under magnetic stirring and refluxed for 24 h. After the reaction was complete, the mixture was cooled to room temperature. The resulting solid was collected by suction filtration through a 0.45 μm membrane filter and washed repeatedly with Milli-Q water until the filtrate pH reached 5 or higher. The resulting white solid was then dried under vacuum at room temperature for 24 h. The synthesis reaction is represented as:

$$ZrO_2 + 2\,H_3PO_4 \rightarrow Zr(HPO_4)_2 \cdot H_2O + H_2O \quad (S1)$$

The crystal structure and thermal properties of the synthesized materials were examined using powder X-ray diffraction (XRD) and differential thermal-thermogravimetric analysis (TG-DTA), as described in Sections 1.4.1 and 1.4.2.

1.2. Adsorption Batch Experiments

The cation-adsorption characteristics of ZrP were evaluated using a batch adsorption method. Sodium (Na^+), potassium (K^+), and ammonium (NH_4^+) ions were selected as the target cation species. A series of cation solutions (10-200 mmol L^{-1}) was prepared by diluting sodium hydroxide (NaOH, FUJIFILM Wako Pure Chemical Corporation, Osaka, Japan, 97%), potassium hydroxide (KOH, FUJIFILM Wako Pure Chemical Corporation, Osaka, Japan, 85%), and ammonium hydroxide solution (NH_4OH aq., FUJIFILM Wako Pure Chemical Corporation, Osaka, Japan, 28-30 wt%) with Milli-Q water.

The batch adsorption procedure is as follows: Approximately 600 mg of ZrP powder was suspended in 30 mL of each cation solution (solid-liquid ratio: 50 mL g^{-1}) and stirred for 6 h in a thermostatic water bath maintained at the designated temperatures (283, 298, 313, and 333 K). After adsorption, solid–liquid separation was performed by suction filtration through a 0.45 μm membrane filter. The filtrate was collected, and the separated solid was rinsed with a small amount of Milli-Q water to remove any residual salts from its surface. The solid samples were then dried under vacuum at room temperature for 24 h. The dried samples were characterized by XRD analysis and TG-DTA to evaluate changes in crystal structure and thermal properties (see Sections 1.4.1 and 1.4.2).

The pH of the filtrate was measured, and the cation concentrations were determined using ion chromatography (IC) (see Section 1.3.1). The amount of cations adsorbed, C_{ab}, was calculated from the concentration difference before and after adsorption using the following equation:

$$C_{\mathrm{ab}} = \{(C_{\mathrm{ini}} - C_{\mathrm{fin}}) \times V \,/\, W_{\mathrm{ab}} \quad (S2)$$

Here, C_{ab} represents the cation adsorption capacity (mmol g^{-1}), C_{ini} and C_{fin} denote the cation

concentrations (mmol L^{-1}) before and after adsorption, respectively, V is the solution volume (L), and W_{ab} is the weight (g) of ZrP used in the experiment. The possible dissolution of the layered ZrP during the adsorption process was examined by measuring the Zr and P concentrations in the filtrate using inductively coupled plasma mass spectrometry (ICP-MS) (see Section 1.3.2 for experimental details).

1.3. Solution Property Characterization

1.3.1. Ion chromatography (IC)

The concentrations of cations (Na^+, NH_4^+, and K^+) in solution were quantified using an IC system (Eco IC, Metrohm AG, Herisau, Switzerland) equipped with a cation-exchange column (Metrosep C4-250/4.0, Metrohm AG, Herisau, Switzerland). The eluent was prepared by dissolving nitric acid (HNO_3, FUJIFILM Wako Pure Chemical Corporation, Osaka, Japan, 68%) and dipicolinic acid ($C_7H_5NO_4$, FUJIFILM Wako Pure Chemical Corporation, Osaka, Japan, ≥98%) in Milli-Q water to achieve final concentrations of 1.7 mM HNO_3 and 0.7 mM $C_7H_5NO_4$. For calibration, a mixed cation standard solution (Na^+, NH_4^+, K^+, FUJIFILM Wako Pure Chemical Corporation, Osaka, Japan, each 100 mg L^{-1}) was diluted with Milli-Q water to prepare standards in the concentration range of 0.01–5 mg L^{-1}. Before measurement, each sample (e.g., filtrate) was diluted with nitric acid and Milli-Q water, as needed, to ensure that the cation concentrations fell within the calibration range and that the sample pH was adjusted between 5 and 8.

1.3.2. Inductively coupled plasma mass spectrometer (ICP-MS)

The concentrations of Zr and P released into the solution during the dissolution of layered ZrP were analyzed using an inductively coupled plasma mass spectrometer (ICP-MS, NexION 2000, PerkinElmer, Inc., Waltham, MA, USA). Indium standard solution (^{115}In, Sigma-Aldrich, St. Louis, MO, USA, 1000 ppb, in 2-3% HNO_3) was used as an internal standard to correct for matrix effects. For calibration, zirconium (^{90}Zr, Sigma-Aldrich, St. Louis, MO, USA, 1000 mg L^{-1} in 7% HCl) and phosphorus (^{31}P, Sigma-Aldrich, St. Louis, MO, USA, 1000 mg L^{-1} in H_3PO_4) standard solutions were diluted with 2% HNO_3 prepared from Milli-Q water and high-purity nitric acid for ICP-MS analysis (TAMAPURE-AA-100, Tama Chemicals Co., Ltd., Kawasaki, Japan, ≥55%) to obtain calibration ranges of 5-50 ppb for ^{90}Zr and 50-500 ppb for ^{31}P.

The limits of quantification (LOQ) for each element were determined using the standard deviation (σ, n = 10) of the blank-corrected intensities and the slope (S) of the calibration curve, according to the following equation:

$$\mathrm{LOQ} = 10\,\sigma / S \tag{S3}$$

The resulting LOQs were 4 ppb for ^{90}Zr (σ = 0.2) and 40 ppb for ^{31}P (σ = 0.04). Before measurement, each sample (e.g., filtrate) was diluted with Milli-Q water as needed to keep the cation concentration below 100 ppm. The natural isotopic abundances of ^{90}Zr and ^{31}P are 0.5145 and 1.000, respectively.

1.4 Solid-State Characterization

1.4.1 Powder X-ray Diffraction (XRD)

The crystal structure and changes in interlayer spacing of the solid samples were evaluated using a powder X-ray diffractometer (XRD, D8 Advance, Bruker Corporation, Billerica, MA, USA) equipped with a Cu Kα radiation source operating at 40 mA and 40 kV.

1.4.2 Thermogravimetric and Differential Thermal Analysis (TG-DTA)

The dehydration behavior associated with cation incorporation was examined using a TG-DTA instrument (Thermo Plus EVO2 TG-DTA8122, Rigaku Corporation, Tokyo, Japan). Measurements were conducted under a nitrogen atmosphere at a heating rate of 5 K min^{-1} over the temperature range from room temperature to 473 K.

1.4.3 Scanning Electron Microscopy with Energy-Dispersive X-ray Spectroscopy (SEM-EDX)

The elemental compositions of the solid samples were evaluated using scanning electron microscopy coupled with energy-dispersive X-ray spectroscopy analysis (SEM-EDX, TM4000Pro, Hitachi High-Tech Corporation, Tokyo, Japan). Prior to analysis, the samples were washed with Milli-Q water following adsorption, desorption, or re-adsorption treatment and dried under vacuum for at least 24 h. Measurements were performed at an accelerating voltage of 15 kV and a magnification of 1000×. The Na/P and K/P molar ratios were derived from the EDX elemental compositions to assess the reversibility of cation exchange.

1.5 Acid Regeneration and Re-adsorption of ZrP

The regeneration of ZrP was evaluated using the batch method. Sodium (Na^+), potassium (K^+) and ammonium (NH_4^+) ions were selected as the target cation species. Each cation solution was prepared by diluting 97% sodium hydroxide (NaOH; FUJIFILM Wako Pure Chemical Corporation, Osaka, Japan), 85% potassium hydroxide (KOH; FUJIFILM Wako Pure Chemical Corporation, Osaka, Japan) or 28-30% ammonium hydrate water (NH_4OH aq., FUJIFILM Wako Pure Chemical Corporation, Osaka, Japan) with Milli-Q water. The acid washing solution was prepared by diluting hydrochloric acid (HCl, FUJIFILM Wako Pure Chemical Corporation, Osaka, Japan, 35–37%) with Milli-Q water.

Approximately 600 mg of ZrP powder was suspended in 30 mL of a 200 mmol L^{-1} cation solution (pH 12; solid-liquid ratio: 50 mL g^{-1}) and stirred at 298 K for 6 h in a thermostatic water bath. After the reaction, the solid and liquid phases were separated by suction filtration through a 0.45 μm membrane filter. The filtrate was collected, and the solid was rinsed with a small amount of Milli-Q water to remove residual salts, then vacuum-dried at room temperature for 24 h. Portions of the dried solid were analyzed using XRD, TG-DTA and SEM-EDX.

Subsequently, approximately 300 mg of the dried white powder was suspended in 15 mL of a 250 mmol L^{-1} hydrochloric acid solution (solid–liquid ratio: 50 mL g^{-1}) and stirred at 298 K for 6 h. The solid was then

recovered by suction filtration using a 0.45 μm membrane filter, rinsed with Milli-Q water, and dried under vacuum at room temperature for 24 h. Portions of the acid-washed powder were again characterized by XRD, TG-DTA and SEM-EDX.

Finally, approximately 200 mg of the acid-washed powder was suspended in 10 mL of a 200 mmol L^{-1} cation solution (solid–liquid ratio: 50 mL g^{-1}) and stirred at 298 K for 6 h. After the reaction, filtration was performed using a 0.45 μm membrane filter. The filtrate was collected for ICP-MS analysis, and the solid was rinsed with Milli-Q water and dried under vacuum at room temperature for 24 h. The recovered solids were subsequently analyzed using XRD, TG-DTA and SEM-EDX.

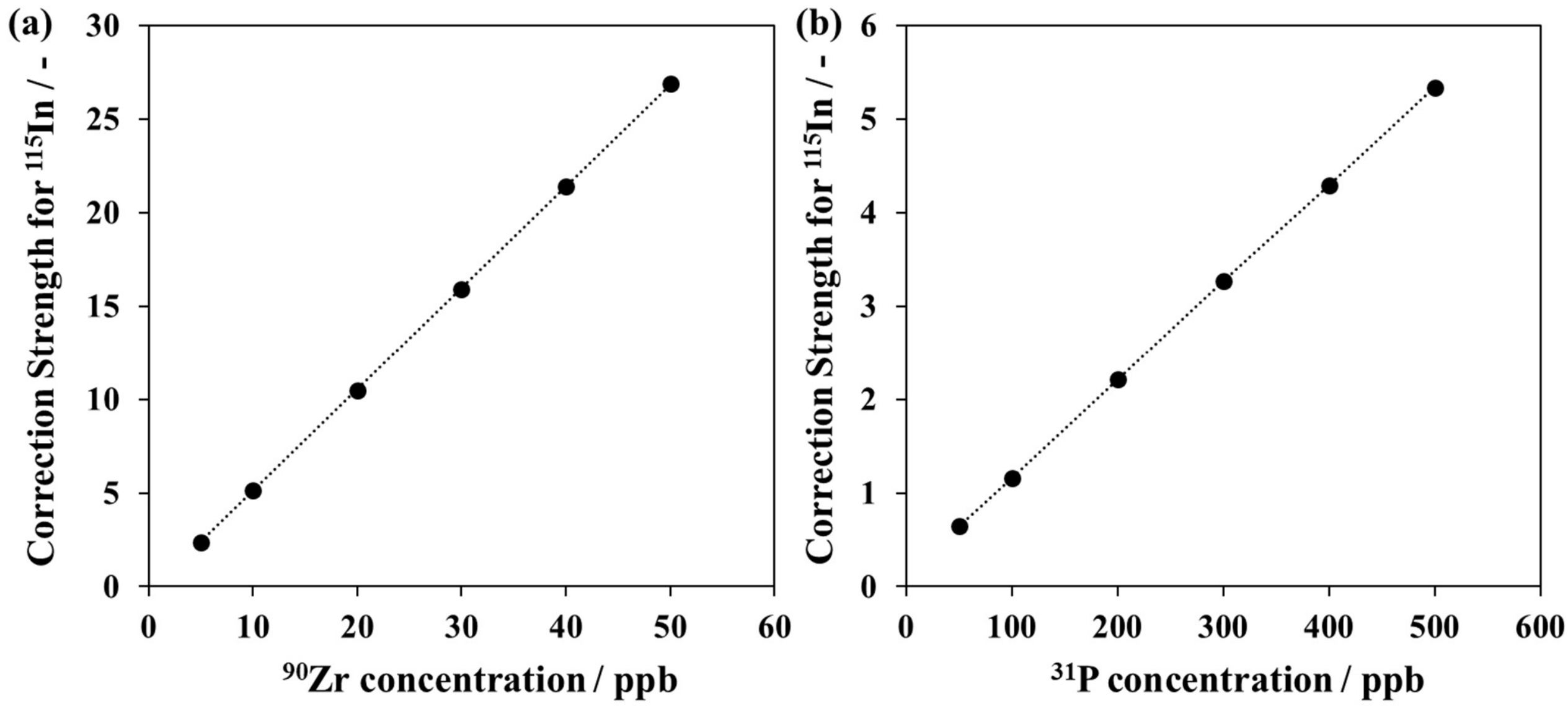

Figure S1. Calculation curves of ICP-MS using ^{115}In, (a)^{90}Zr and (b) ^{31}P

Table S1. Calibration parameters for ICP-MS analysis of ^{90}Zr and ^{31}P.

Element	Calibration range (ppb)	Slope	Intercept	R^2	LOQ (ppb)
^{90}Zr	5–50	0.54369	-0.35423	0.99998	4
^{31}P	50–500	0.01043	0.12272	0.99999	40

Table S2. Na/P and K/P molar ratios determined by SEM-EDX analysis.

	Na/P mol rate		K/P mol rate
ZrP only	0.00	ZrP only	0.00
2 $mol_{Na}\ mol_{ZrP}^{-1}$	1.18	2 $mol_{K}\ mol_{ZrP}^{-1}$	1.01
Desorbed	0.00	Desorbed	0.03
Reabsorbed	1.25	Reabsorbed	1.00
